# Travel Vaccines Enter the Digital Age: Creating a Virtual Immunization Record

**DOI:** 10.4269/ajtmh.15-0510

**Published:** 2016-03-02

**Authors:** Kumanan Wilson, Katherine M. Atkinson, Cameron P. Bell

**Affiliations:** Clinical Epidemiology Program, Ottawa Hospital Research Institute, Ottawa, Ontario; Department of Medicine, University of Ottawa, Ottawa, Ontario; Department of Epidemiology and Community Medicine, University of Ottawa, Ottawa, Ontario; Department of Public Health Sciences, Karolinska Institutet, Stockholm, Sweden

## Abstract

At present, proof of immunization against diseases such as yellow fever is required at some international borders in concordance with the International Health Regulations. The current standard, the International Certificate of Vaccination or Prophylaxis (ICVP), has limitations as a paper record including the possibility of being illegible, misplaced, or damaged. We believe that a complementary, digital record would offer advantages to public health and travelers alike. These include enhanced availability and reliability, potential to include lot specific information, and integration with immunization information systems. Challenges exist in implementation, particularly pertaining to verification at border crossings. We describe a potential course for the development and implementation of a digital ICVP record.

## Background

Vaccination is one of the most effective mechanisms to prevent the global spread of infectious diseases. To be successful in this capacity, international vaccination requirements must be established as is the case for jurisdictions with risk of transmission for yellow fever and most recently, polio.^1^ At present, travelers must present authenticated paper records as proof of immunization at border crossings when traveling through these areas. However, paper records can be difficult to read and are easily misplaced or damaged. As the world embraces mobile technologies and smartphone use becomes increasingly prevalent, an opportunity exists to develop a digital record for proof of immunization. We believe an authenticated digital representation of ones' vaccination records for travel could serve as a useful complement to existing paper-based record keeping and immunization information systems around the world.^2^,^3^

## Proof of Vaccination

The International Health Regulations (IHRs) are the primary document that governs international preparation and response to public health emergencies that can cross borders. Annex 7 of the 2005 revisions to the Regulations (IHR 2005) identifies yellow fever as a condition for which proof of vaccination may be necessary if arriving from an affected area as designated by the World Health Organization (WHO).^4^ Failure to present proof of vaccination can result in quarantine for up to 6 days, refused entry, or vaccination on site.^5^–^7^ Under the authority to declare a public health emergency of international concern, the IHR (2005) also empowers the Director General of the WHO to issue temporary recommendations informed by the advice of an emergency committee, which can include requirements for vaccination.^8^ Such a scenario unfolded in 2014 with the declaration of polio as a public health emergency of international concern and the requirement for proof of polio vaccination for people traveling from areas exporting polio cases.^9^ In February 2015, an extension of these recommendations was unanimously endorsed. As such individuals traveling from Cameroon, Equatorial Guinea, Syrian Arab Republic, and Pakistan where, at the time, governed by this legislation requiring travelers leaving these jurisdictions to provide an International Certificate of Vaccination or Prophylaxis (ICVP) when arriving at borders.^10^

## Current Solution

Proof of vaccination currently requires an authenticated paper record. Annex 6 of the IHR (2005) presents the model WHO/IHR “yellow booklet” ICVP. The validity of this certificate is dependent on the documentation of the vaccination or prophylaxis, date of receipt, signature and status of clinician (by hand), manufacturing and batch number, expiry date of certificate, and official stamp of administering center.^11^

Although the paper record approach has the advantages of simplicity, authenticity, and universality, it also has limitations. The first and perhaps most important limitation is ensuring consistent and reliable access to the record. Even when meticulously prepared and perfectly legible at the time of documentation, paper records are easily misplaced and are vulnerable to damage, potentially even to the extent of compromising their legibility and validity. This could be particularly problematic for individuals who need to urgently travel, for example, business travelers. The time to retrieve or create replacement records could serve as an obstacle to travel and could potentially dissuade entities from engaging in commerce in affected jurisdictions. In scenarios where individuals travel with family or young children, the absence of access to records for all parties could also serve as an impediment to travel. This also creates risk of unnecessary repeat vaccinations because of lack of certainty regarding the date of vaccination or absence of documentation entirely.

The explicit purpose of the IHR (2005) is “to prevent, protect against, control and provide a public health response to the international spread of disease in ways that are commensurate with and restricted to public health risks, and which avoid unnecessary interference with international traffic and trade.”^5^–^7^,^12^ The requirement for a paper record for proof of vaccination could be viewed as “unnecessary interference with international travel and trade” if a more accessible option were also available.

## Digital Records

There now exists more mobile devices in the world than people, representing the fastest adoption of a manufactured technology in history.^13^ The rapid integration of these technologies has generated novel opportunities to improve many facets of our everyday lives. The field of health is no exception. Mobile health or “mHealth” has been regarded as a game changer, empowering individuals and helping the system to deliver higher quality at a reduced cost.^14^–^16^

The promise of mobile health is also true for public health. For example, mobile telephones have demonstrated the capacity to improve multiple aspects of immunization practice, including increasing vaccination coverage.^17^–^19^

**Figure 1.**
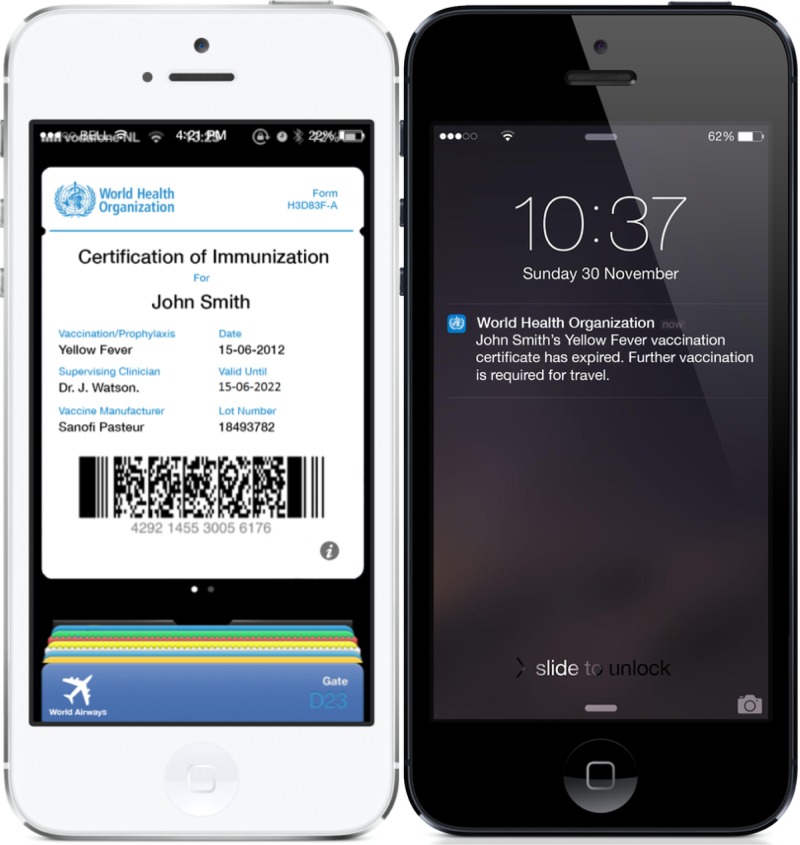
Screenshots of the proposed digital solution.

Thus, we believe that the potential exists to create a complementary, digital version of the ICVP (Figure 1

). Such a record could incorporate all of the elements of the current ICVP as well as others that are unique to digital systems. A complementary digital solution could offer additional advantages to travelers and public health alike. Some of these are mentioned below.

### Increased availability and reliability of records.

A user would have access to a record of the immunization on their device with the option to store in a centralized repository. This centralized repository would allow for remote access to one's records over the Internet and facilitate synchronization of data should the user lose or replace their device.

### Improved authenticity.

**Figure 2.**
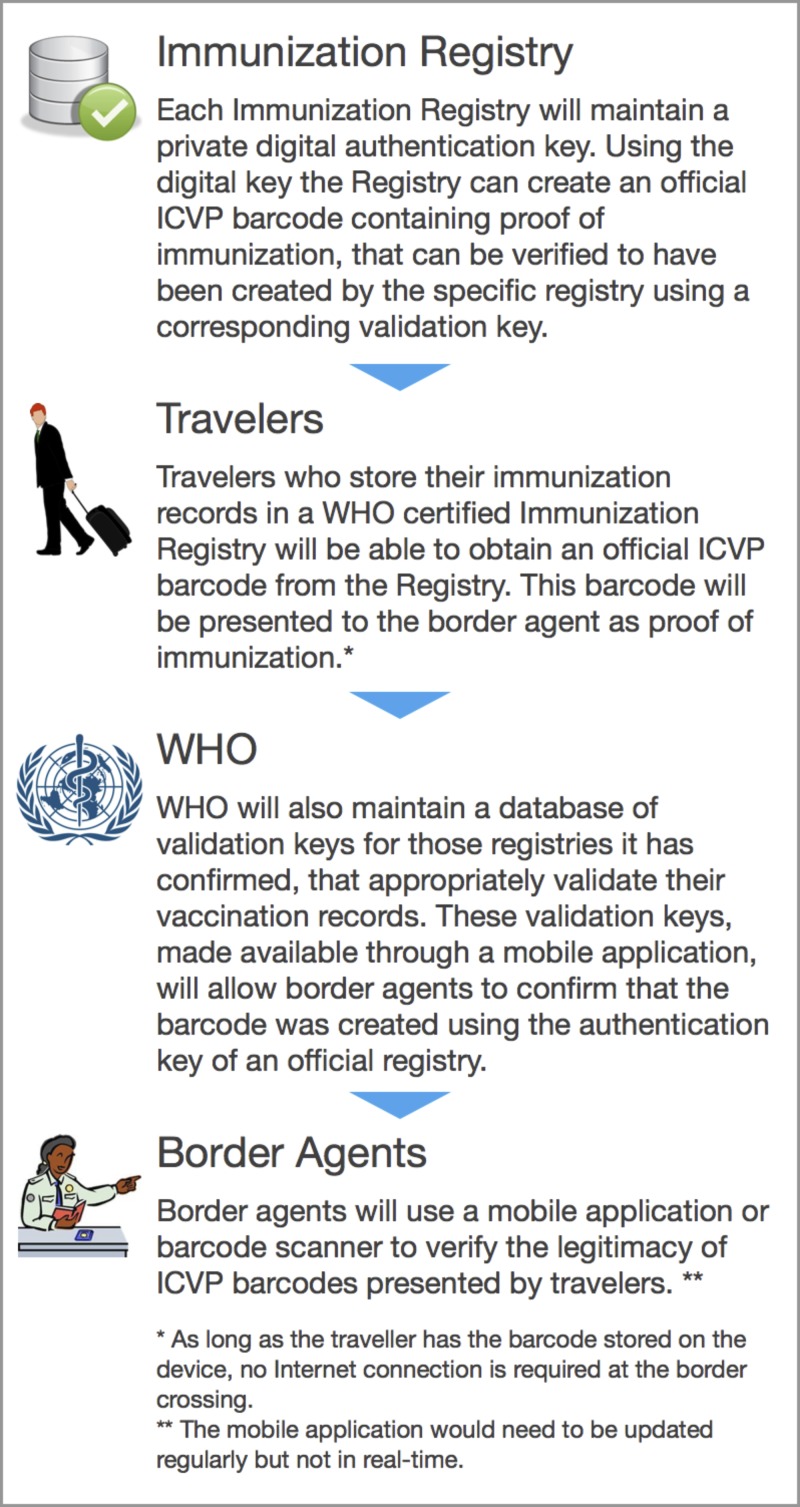
Implementation of a digital ICVP.

Properly implemented digital authentication schemes may be less prone to forgery than traditional ink stamps on paper. We describe one approach, using digital authentication key pairs, to validate digital ICVPs in Figure 2

.

### Vaccination reminders.

Mobile devices offer the potential to provide passive reminders when the expiry of vaccination information is approaching, directly through the individual's mobile device. This could also be linked to appointment booking functionality with the administrator or a “vaccine finder” service such as HealthMap's Vaccine Finder (http://vaccine.healthmap.org/).^20^

### Lot-specific information.

Smartphones introduce the potential to scan a bar code and instantly upload the lot-specific information of the vaccine administered into the record. Digital upload of bar coded data has been shown to reduce the transcription errors and recording time.^21^ Access to lot-specific information also offers utility for vaccine safety surveillance and supply chain management.

### Automated identification.

Bar codes presented on the screen of a mobile device can permit rapid data transfer between devices, as seen in modern airport ticketing systems. This would enable rapid validation of one's record at a border crossing.

## Challenges to Implementation

Although a complimentary digital version has advantages, there also exist some potential limitations. Perhaps most challenging is the adoption of the system by validators, who are familiar with the paper-based process. If a digital version of the record was created, it would need to have a globally accepted mechanism to authenticate the record, as is in place with paper records now via stamps in the United States. Many digital authentication schemes require access to the Internet to assess the validity of a digital signature. This would be a challenge in remote areas that may not have reliable Internet access. Therefore, adequate technical infrastructure in addition to education and training for jurisdictions prior to implementation would be crucial to success.

In addition to digital authentication schemes, we believe that authentication could be enhanced through other options such as unique provider personal identification number (PIN) entry adjacent to a signature for matching or use of biometrics such as fingerprint scanners present on new smartphones. We envision the digital solution to be complementary to, not replacing, the paper-based solution. A phase-in approach may be necessary where a paper record is required and the digital record may be used for convenience, until there is sufficient adoption to allow for the use of the digital record isolation. Thus, smartphone users and those with web access, which we expect would be a high percentage of business travelers, would have access to this option that could facilitate travel. This would provide an opportunity to identify and conquer barriers to implementing globally interoperable digital health solutions.

## Implementation

To implement a digital record of the proof of vaccination record, we would suggest the following (Figure 2):

1. Key stakeholders (e.g., WHO, national governments, regulatory agencies, travel industry, vaccine manufacturers, and travel physicians) would need to arrive at a consensus as to what elements of a vaccination record would need to be created digitally and what mechanisms to enable this would be considered acceptable.

The impact any solution would have on current work-flow practices at administration centers and borders would need to be evaluated.

2. Development of a prototype for a digital proof of vaccination for use in one jurisdiction.
3. Demonstrate integration within a new or existing mobile application.

Delivering this service within a broader mobile product has the potential to combine this functionality while creating a direct channel to users. This channel could be leveraged to send alerts and deliver tailored information. We have developed a national immunization record for Canada, ImmunizeCA.^22^ A digital record could be incorporated into this app or kept separate, such as in the Passbook app for iOS devices.

4. Explore the potential to create centralized immunization data systems for travel vaccinations that could be accessible to travelers and border officials.

## Conclusion

Mobile devices provide an opportunity to reduce impediments to international travel and trade while upholding surveillance and containment of disease in endemic and transitional jurisdictions. Failure to modernize public health practices, such as a paper-based ICVP, introduces liabilities to these countries, which may already be vulnerable, both from population health and economic sustainability perspectives.
